# Soil phosphorus form affects the advantages that arbuscular mycorrhizal fungi confer on the invasive plant species, *Solidago canadensis*, over its congener

**DOI:** 10.3389/fmicb.2023.1160631

**Published:** 2023-04-14

**Authors:** Li Chen, Mengqi Wang, Yu Shi, Pinpin Ma, Yali Xiao, Hongwei Yu, Jianqing Ding

**Affiliations:** ^1^State Key Laboratory of Crop Stress Adaptation and Improvement, School of Life Sciences, Henan University, Kaifeng, China; ^2^College of Life Sciences, Jiangxi Science and Technology Normal University, Nanchang, China; ^3^School of Life Sciences and Agricultural Engineering, Nanyang Normal University, Nanyang, China

**Keywords:** Glomeraceae, keystone taxa, microbial diversity, nutrient form, plant invasion, plant-fungal interaction

## Abstract

Interactions between plants and arbuscular mycorrhizal fungi (AMF) are strongly affected by soil phosphorus (P) availability. However, how P forms impact rhizosphere AMF diversity, community composition, and the co-occurrence network associated with native and invasive plants, and whether these changes in turn influence the invasiveness of alien species remain unclear. In this work, we performed a greenhouse experiment with the invasive species *Solidago canadensis* and its native congener *S. decurrens* to investigate how different forms of P altered the AMF community and evaluate how these changes were linked with the growth advantage of *S. canadensis* relative to *S. decurrens*. Plants were subjected to five different P treatments: no P addition (control), simple inorganic P (sodium dihydrogen phosphate, NaP), complex inorganic P (hydroxyapatite, CaP), simple organic P (adenosine monophosphate, AMP) and complex organic P (myo-inositol hexakisphosphate, PA). Overall, invasive *S. canadensis* grew larger than native *S. decurrens* across all P treatments, and this growth advantage was strengthened when these species were grown in CaP and AMP treatments. The two *Solidago* species harbored divergent AMF communities, and soil P treatments significantly shifted AMF community composition. In particular, the differences in AMF diversity, community composition, topological features and keystone taxa of the co-occurrence networks between *S. canadensis* and *S. decurrens* were amplified when the dominant form of soil P was altered. Despite significant correlations between AMF alpha diversity, community structure, co-occurrence network composition and plant performance, we found that alpha diversity and keystone taxa of the AMF co-occurrence networks were the primary factors influencing plant growth and the growth advantage of invasive *S. canadensis* between soil P treatments. These results suggest that AMF could confer invasive plants with greater advantages over native congeners, depending on the forms of P in the soil, and emphasize the important roles of multiple AMF traits in plant invasion.

## Introduction

Plant invasions are a serious threat to biodiversity and ecosystem functions, particularly in recent decades due to globalization (Livingstone et al., 2020; Rai and Singh, 2020). The invasive success of exotic species is complex to predict and depends on a range of biotic and abiotic conditions (Enders et al., 2020). Of all the factors affecting the success of plant invasions, plant association with soil mutualistic microbes contributes greatly to the performance of exotic species by promoting nutrient absorption and tolerance to stressors (Reinhart and Callaway, 2006; Lamit et al., 2022). However, the magnitude and direction of symbiotic associations between plants and soil microbes are frequently affected by soil nutrients (Chen et al., 2020, 2021). Thus, greater attention should be paid to soil nutrient mediated interactions between invasive plants and mutualistic microbes. Insights into soil mutualistic microbes associated with invasive plants under various nutrient conditions could help improve understanding of the microbiome-related mechanisms underlying invasion success.

Arbuscular mycorrhizal fungi (AMF) establish symbiotic relationships with most terrestrial plants and provide multiple benefits to host plants (Basu et al., 2018; Mathur et al., 2019; Cao et al., 2020). Consequently, shifts in the AMF community and their interconnections may influence plant growth and fitness (Liang et al., 2019; Schröder et al., 2019). For example, a higher AMF richness or greater abundance of specific AMF taxa may enhance plant productivity and promote plant diversity (Vogelsang et al., 2006), and plant species that harbor distinct AMF communities have been shown to exhibit heterogenous growth responses (Sheng et al., 2022). Moreover, the effects conferred by mycorrhiza may vary between native and invasive species. Awaydul et al. (2019) found that mycorrhizal networks increased nutrient acquisition in invasive plants compared with native plants. Therefore, variations in AMF diversity, community structure, and co-occurrence networks could potentially affect plant growth and the invasiveness of exotic plants. Although a growing number of studies have highlighted the importance of AMF to invasion success of exotic plants (Majewska et al., 2017; Yu et al., 2022), the roles of AMF taxa, community assembly, and their relative contributions to the success of plant invasions are still poorly understood.

Phosphorus (P) is a growth-limiting nutrient in many natural ecosystems, playing important roles in plant and AMF growth and metabolism. For example, soil P can affect invasive plant performance by changing functional traits or photosynthesis (Esterhuizen et al., 2020). P can also significantly mediate AMF spore growth (UI Haq et al., 2022), colonization rate (Grman and Robinson, 2013), hyphal length (Xiang et al., 2014) and community structure (Wang et al., 2022). Thus, the key role of soil P availability in mediating the invasive plant-AMF interaction is well accepted (Chen et al., 2020). However, soil P exists in a range of inorganic and organic compounds (Song et al., 2007; Azene et al., 2022), and different forms of P differ distinctly in their efficacy on invasive plant growth and AMF metabolism and functions (Yang et al., 2020; Qi et al., 2022; Zhang et al., 2022). Additionally, existing evidence regarding soil nutrient-modulated plant-AMF interactions has typically focused on mycorrhizal colonization or hyphal density (Chen et al., 2020; Ma et al., 2020), while ignoring mycorrhizal response may also depend on complex microbial interactions (Chaudhary et al., 2022). Therefore, investigating the effect of different forms of P on AMF communities and identifying the essential components of mycorrhizal traits that enhance invasive plant performance could provide mechanistic insights into soil nutrient-regulated plant-fungal interactions, which are crucial to the management of invasive plants.

*Solidago canadensis*, one of the most notorious invasive plants in the world, was introduced into China in the 1930s (Dong et al., 2006a). In the introduced range, *S. canadensis* was characterized by rapid growth and high reproductive capacity (Dong et al., 2006b) and formed strong mutualistic interactions with AMF (Yu and He, 2022), which helped it outcompete the native species. Invasive *Solidago* can maintain its dominance across a range of soil nutrient environments (Dong et al., 2006a), including inorganic P-dominant and organic P-dominant habits (Yang et al., 2020). Although previous studies found that *S. canadensis* can benefit more from organic than inorganic P (Yang et al., 2020; Zhang et al., 2022) and perform better compared with the native species *S. decurrens* under diverse nutrient conditions (Yu and He, 2022), few studies have explicitly tested whether this is associated with AMF community.

In this study, we conducted a greenhouse experiment to examine the impacts of different forms of P in the soil on the AMF community, and evaluate their effects on the growth and growth advantage of *S. canadensis*. We hypothesized that: (1) native and invasive species would respond differently to different forms of P in terms of AMF diversity, community structure, co-occurrence network, and plant performance; (2) differences in AMF traits conferred by different soil P environments contribute differently to plant growth and growth advantage in *S. canadensis*. Our work provides clear evidence that soil P conditions alter multiple mycorrhizal traits, which is linked to enhanced invasive plant performance.

## Materials and methods

### Study species

*Solidago canadensis* L., native to North America, is a perennial forb propagated by seeds and rhizomes. Since its introduction into China in 1935 (Dong et al., 2006a), *S. canadensis* has invaded large areas and diverse habitats in southern China (Dong et al., 2006a; Yu and He, 2022). *Solidago decurrens* L., a native congener perennial forb, occurs commonly in the range that was invaded by *S. canadensis* in China. We collected *S. canadensis* and *S. decurrens* seeds from natural populations and used them for the following experiment.

### Experimental design

The experiment was conducted in a greenhouse from May to August at Henan University campus, Kaifeng, China (34°49′13′′ N, 114°18′ 18′′ E). We collected soil from barren land and sieved it through 2 mm mesh to remove roots and other debris. To prepare the growth medium, we mixed the soil with an equal volume of sand to minimize the potential adverse effects of excessive P in the soil. The basic properties of this growth medium were as follows: 11.5 ± 0.13 g·kg^−1^ carbon, 0.1 ± 0.01 g·kg^−1^ total nitrogen, 3.8 ± 0.21 mg·kg^−1^ available nitrogen, 1.4 ± 0.15 mg·kg^−1^ available phosphorus, and pH of 7.9 ± 0.01. The growth medium was divided into 2 kg portions in individual 2 l pots (11 cm × 16 cm × 14 cm).

To investigate the effects of different P sources on plant performance and rhizosphere AMF communities, we set up five P treatments: control (no P addition), sodium dihydrogen phosphate (NaH_2_PO_4_, simple inorganic P, NaP), hydroxyapatite [Ca_5_(OH)(PO_4_)_3_, complex inorganic P, CaP], adenosine monophosphate (C_10_H_14_N_5_O_7_P, simple organic P, AMP), and myo-inositol hexakisphosphate (C_6_H_18_O_24_P_6_, complex organic P, PA). We designed these P treatments based on previous research (Pearse et al., 2007; Yang et al., 2020; Qi et al., 2022). The P concentration was supplied at 20 mg kg^−1^ soil, roughly corresponding to the medium soil P content in areas invaded by invasive plants (Chen et al., 2020). We supplemented the four P sources to pots and mixed them with growth medium thoroughly.

Seeds of *S. canadensis* and *S. decurrens* were surface-sterilized with 2% NaClO for 2 min, then germinated in trays filled with 25 kGy gamma-irradiation sterilized vermiculite. When seedlings reached the three-leaf stage, similar size seedlings were transplanted into the pots as described above. All the pots were positioned randomly in the greenhouse and rotated each week to avoid the potential effects of microsite variability. The growth conditions in the greenhouse were as follows: 16 h light (day) and 8 h dark (night), 25°C during the day and 18°C during the night at a relative humidity of 60%. To avoid other nutrient limitations on plant growth, we supplemented equivalent modified P-free Hoagland’s nutrient solution to each pot during the course of experiments. Plants were watered daily to ensure that all plants had sufficient water for growth. Each treatment was replicated eight times, resulting in 80 pots in total.

### Plant harvest, soil sampling, and calculation of relative change in biomass

After 70 days growth, the experimental plants were harvested, dried at 60°C for 72 h, and weighed to determine whole-plant biomass. To evaluate difference in plant performance between native and invasive species, we calculated the growth advantage (GA) of invasive *S. canadensis* over its native conger *S. decurrens* according to the following equation:


GA=[(Sc−Sd)/Sd]×100%

where Sc and Sd are the biomass of *S. canadensis* and *S. decurrens*, respectively, grown in a given P treatment. The growth advantage can signify an invader’s potential invasiveness, and a high GA value indicates high plant invasiveness.

Rhizosphere soil samples were collected when the plants were harvested. At harvest, we removed the whole plant from the soil, gently shook the plant to remove the loosely adhering soil around the roots, and collected the tightly adhering soil around the root (rhizosphere soil) by manually brushing (Shi et al., 2022). In total, 80 rhizosphere soil samples were collected. All of these samples were frozen at −80°C for DNA extraction within 2 weeks.

### DNA extraction, polymerase chain reaction amplification, and bioinformatic analyzes

Soil microbial DNA was extracted from 0.25 g soil of each soil sample using Magabio Soil and Feces Genomic DNA Purification Kit (Bioer Technology, Zhejiang, China) according to the manufacturer’s instructions; DNA concentration and purity were then assessed using a NanoDrop One (Thermo Fisher Scientific, MA, United States). The small subunit rRNA (SSU rRNA) region was amplified using the primer set AMV4.5NF (5′-AAGCTCGTAGTTGAATTTCG-3′) and AMDGR (5′-CCCAACTATCCCTATTAATCAT-3′) (Lumini et al., 2010). The polymerase chain reaction (PCR) reactions, containing 25 μl 2x Premix Taq, 1 μl each forward and reverse primer (10 μM), and 3 μl DNA template (20 ng/μl) in a volume of 50 μl, were amplified by thermocycling: 5 min at 94°C for initialization; 30 cycles of 30 s denaturation at 94°C, 30 s annealing at 52°C, and 30 s extension at 72°C; and 10 min final elongation at 72°C. PCR products were quality-checked using 1% agarose gel electrophoresis, and purified with E.Z.N.A. Gel Extraction Kit (Omega, USA). Finally, equimolar concentrations of amplified samples were pooled and sequenced using an Illumina Nova 6000 platform at Magigene Biotechnology Co., Ltd. (Guangzhou, China).

QIIME2 (Bolyen et al., 2019) was used to process paired-end sequence reads. Using the q2-dada2 plugin, raw sequence data were quality-filtered and de-replicated, chimeras were removed, and sequences were grouped into amplicon sequence variations (ASVs) (Callahan et al., 2016). For taxonomic classification, representative sequences were blasted against the MaarjAM database using the q2-feature-classifier plugin, with at least 95% query coverage and 97% sequence identity (Frew, 2022). Non-AMF sequences and singletons were excluded prior to the analysis. The resultant dataset was rarefied at the minimum number of sequences across all samples, and the data were used for the downstream analysis. All sequence data were archived in the NCBI Sequence Read Archive (SRA) database under accession number PRJNA922727.

### Arbuscular mycorrhizal fungi co-occurrence network construction

Network analyzes of the AMF ASVs in the two *Solidago* species under different P treatments were conducted individually. Co-occurrence networks were constructed using the SparCC method on the iNAP platform through the publicly available pipeline^1^ (Feng et al., 2022). Based on 20 iterations and 100 bootstraps, pair-wise ASVs with strong correlations (*r* > 0.5 and *p* < 0.05) were retained to construct the networks. Based on the within-module connectivity (*Zi*) and among-module connectivity (*Pi*) values, we divided the nodes in each network into four categories: peripherals (*Zi* < 2.5 and *Pi* < 0.62, nodes with few links and play a negligible role in the network), connectors (*Zi* < 2.5 and *Pi* > 0.62, link modules and crucial to network coherence), module hubs (*Zi* > 2.5 and *Pi* < 0.62, link nodes within a module that are vital to the module) and network hubs (*Zi* > 2.5 and *Pi* > 0.62, link nodes both within and among modules that are important to the network). This allowed us to evaluate potential topological roles of taxa in the networks. Nodes serving as hubs or connectors in a network were defined as keystone species in the network. The topological properties were analyzed and the networks were visualized using Gephi v0.9.3 (Bastian et al., 2009).

### Statistical analysis

All statistical analyzes were performed using R v4.1.2 (R Core Team, 2022) using the agricolae, (Mendiburu, 2021) vegan (Oksanen et al., 2022), and plspm (Sanchez et al., 2017) packages. A two-way analysis of variance (ANOVA) was conducted to evaluate the effects of plant species and P treatments on plant biomass, alpha diversity, and relative abundance of AMF families. One-way ANOVA with least significant difference (LSD) *post-hoc* tests were implemented when significant differences among treatments were detected. Independent *t*-tests were further used to examine the difference in biomass and alpha diversity between native and invasive *Solidago* species under the same P treatment. Data were square root or natural log transformed to meet the assumption of normality. To assess variation in AMF community composition, principal coordinate analysis (PCoA) based on Bray–Curtis distance matrices and permutational multivariate analysis of variance (PERMANOVA) were used to describe the significance of plant species and P forms on microbial composition. Pairwise comparisons were used to compare differences between different P treatments on AMF community composition. Venn analysis was performed to identify the number of unique and shared ASVs among the five P treatments in the native and invasive *Solidago*. Relationships between AMF variables (i.e., alpha diversity, relative abundance at the family level, and abundance of keystone species in the networks) and plant performance (i.e., biomass and the growth advantage of *S. canadensis*) were evaluated using Pearson correlation.

Partial least squares path modeling (PLS-PM) was performed to quantify the influence of P treatments on plant growth and the growth advantage of *S. canadensis* conferred by the AMF. PLS-PM analyzes were carried out according to previously published methods (Sanchez, 2013). We defined P treatments, alpha diversity, community structure, keystone species, total biomass, and growth advantage as latent variables, and assumed that P treatments affected total biomass or growth advantage *via* alpha diversity, community structure, and keystone species in the co-occurrence networks. Only variables with significant relationships to biomass were included in the models. Based on biomass, we defined and descended the P treatments as integers from 5 to 1. Dillon-Goldstein’s rho was used to evaluate how well a block of indicators described their corresponding latent construct. Loadings reflected the correlations between a latent variable and its indicators. The Dillon-Goldstein’s rho and loadings greater than 0.7 were defined as acceptable (Supplementary Table S1). To evaluate the quality of the model, goodness-of-fit (GoF) and *R*^2^ determination coefficients were calculated. Moreover, we assessed the relative contribution of each latent variable to the R^2^ of total biomass and the growth advantage of *S. canadensis* using the following equation:


$$R^{2}\left(\%\right)=\beta_{j}cor\left(y,x_{j}\right)/\underset{j}{\sum }\beta_{j}cor\left(y,x_{j}\right)$$

where j is the number of latent variables, β is the path coefficient, and cor(y, x_j_) is the correlation between explanatory and response variables (Tenenhaus et al., 2005; Yu and He, 2017).

## Results

### Effects of different forms of P on plant performance

**Figure 1:**
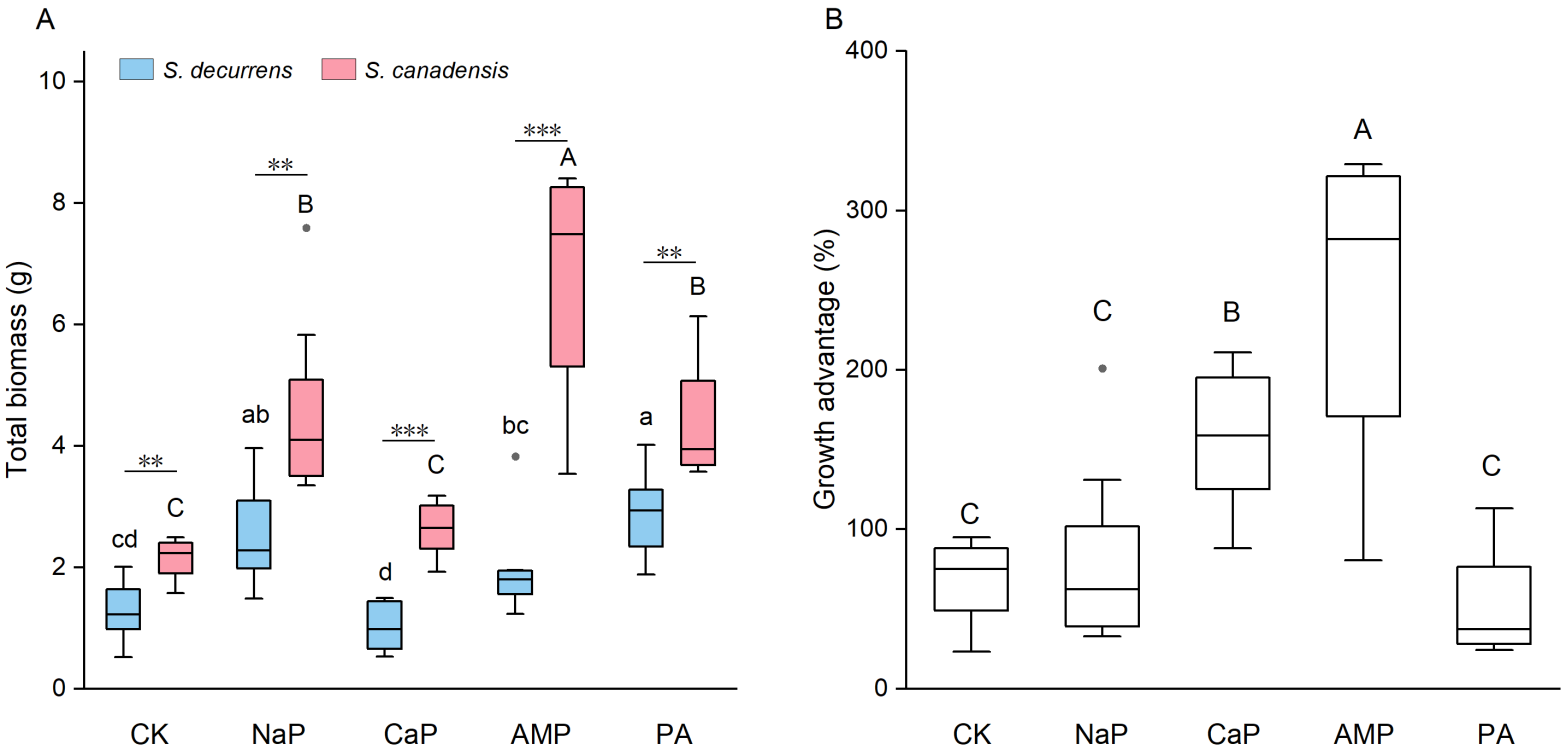
**(A)** Total biomass of *S. decurrens* and *S. canadensis* grown under five P treatments. CK: no P addition; NaP: sodium dihydrogen phosphate; CaP: hydroxyapatite; AMP: adenosine monophosphate; PA: myo-inositol hexakisphosphate. The line in the box represents the median value, box boundaries indicate the value in the 25–75th percentile range, whiskers indicate the 95% confidence intervals. The different lower-case letters among *S. decurrens* and different upper-case letters among *S. canadensis* denote significant differences at *p* < 0.05. The asterisks denote difference between *S. decurrens* and *S. canadensis* in the same P treatment. *p < 0.05, **p < 0.01, ***p < 0.001. **(B)** The growth advantage of *S. canadensis* over *S. decurrens* under five P treatments. Different upper-case letters above the boxes indicate significant differences at *p* < 0.05.

Overall, the invasive *S. canadensis* grew larger than the native *S. decurrens* across all treatments (4.10 ± 0.32 g vs. 1.93 ± 0.15 g; *F*_S_ = 128.51, *p* < 0.001). P treatments, as well as their interactions with the plant species, exerted significant effects on whole-plant biomass (all *p* < 0.001, Figure 1A). Specifically, NaP and PA increased whole-plant biomass in the native *S. decurrens* while the supplementation of NaP, AMP, and PA promoted the growth of the invasive *S. canadensis* (Figure 1A). Compared with *S. decurrens*, *S. canadensis* had greater biomass under each of the five P treatments, leading to a substantial growth advantage for invasive *Solidago*. However, the growth advantage of *S. canadensis* varied with P forms (*F*_P_ = 16.01, *p* < 0.001) and was enhanced with application of CaP and AMP (Figure 1B).

### Arbuscular mycorrhizal fungi diversity and its relationship with plant performance

**Figure 2:**
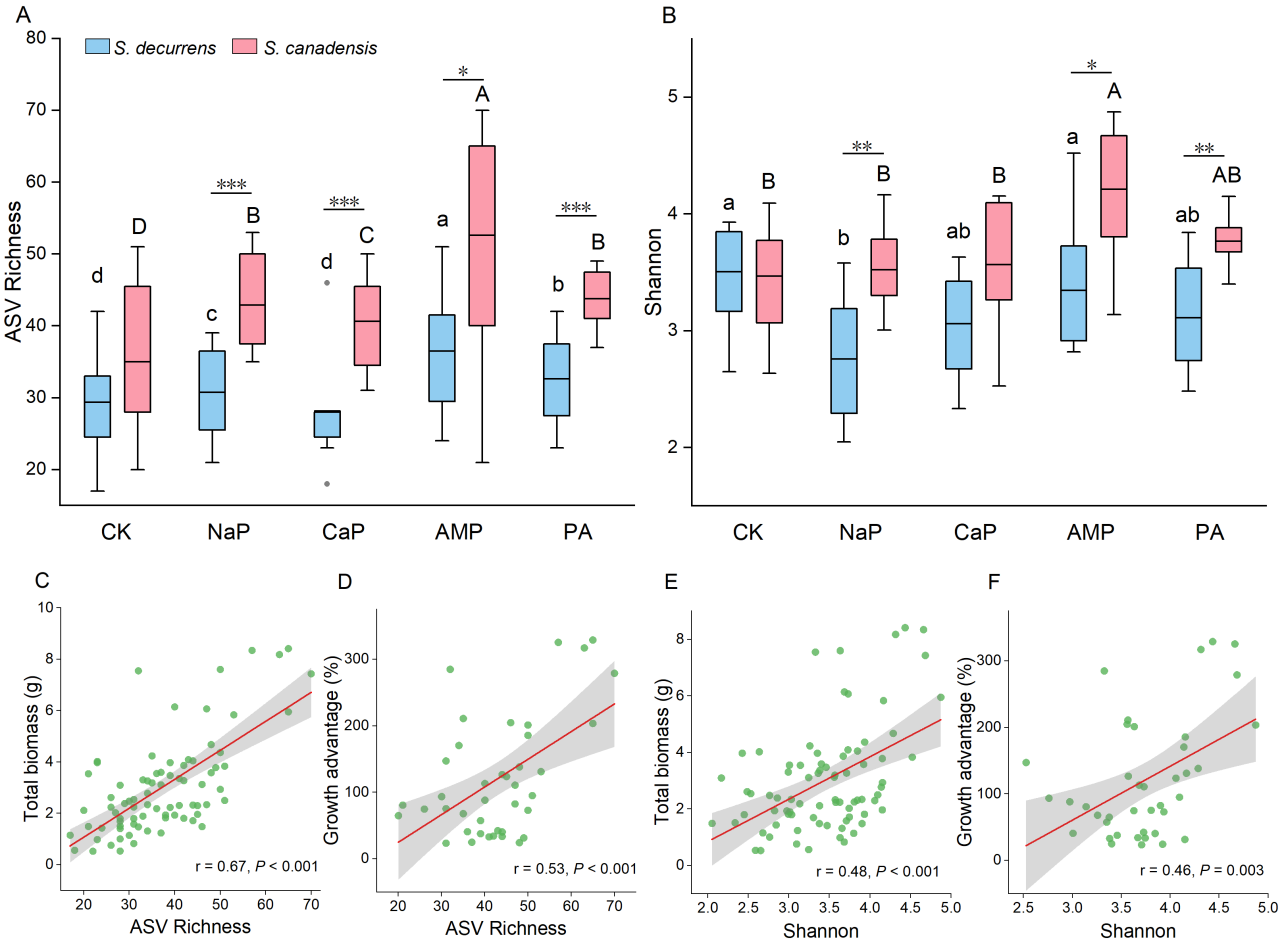
The variation of arbuscular mycorrhizal fungi (AMF) **(A)** ASV richness and **(B)** Shannon index in the rhizosphere soil of *S. decurrens* and *S. canadensis* under five P treatments. CK: no P addition; NaP: sodium dihydrogen phosphate; CaP: hydroxyapatite; AMP: adenosine monophosphate; PA: myo-inositol hexakisphosphate. The line in the box represents the median value, box boundaries indicate values in the 25–75th percentile range, whiskers indicate the 95% confidence intervals. The different lower-case letters among *S. decurrens* and different upper-case letters among *S. canadensis* denote significant differences at *p* < 0.05. The asterisks denote difference between *S. decurrens* and *S. canadensis* in the same P treatment. *p < 0.05, **p < 0.01, ***p < 0.001. Pearson correlations between ASV richness and **(C)** plant growth, **(D)** growth advantage of *S. canadensis* over *S. decurrens*, Shannon index and **(E)** plant growth, **(F)** growth advantage of *S. canadensis* over *S. decurrens*. The gray ribbons represent the 95% confidence intervals.

In total, 1,568,984 high-quality AMF sequence reads were produced before rarefication, with 528 ASVs identified from all soil samples. After rarefication, the AMF alpha diversity was strongly influenced by plant species and P treatments. Across all treatments, *S. canadensis* had a greater richness (*F*_S_ = 33.57, *p* < 0.001) and Shannon index (*F*_S_ = 26.77, *p* < 0.001) than *S. decurrens* (Figures 2A,B). Richness and Shannon index were similar between the two species in the control treatment (Figures 2A,B). However, there were clear differences in alpha diversity between the two *Solidago* species when P was added to the soil, with the invasive *Solidago* showing a higher response to resource fluctuations in terms of microbial diversity (Figures 2A,B). Of all P treatments, AMP treatment resulted in the highest richness and Shannon index for both species (Figures 2A,B). Furthermore, the whole-plant biomass and the growth advantage of *S. canadensis* increased with the increasing alpha diversity (Figures 2C–F).

### Arbuscular mycorrhizal fungi community structure and its relationship with plant performance

**Figure 3:**
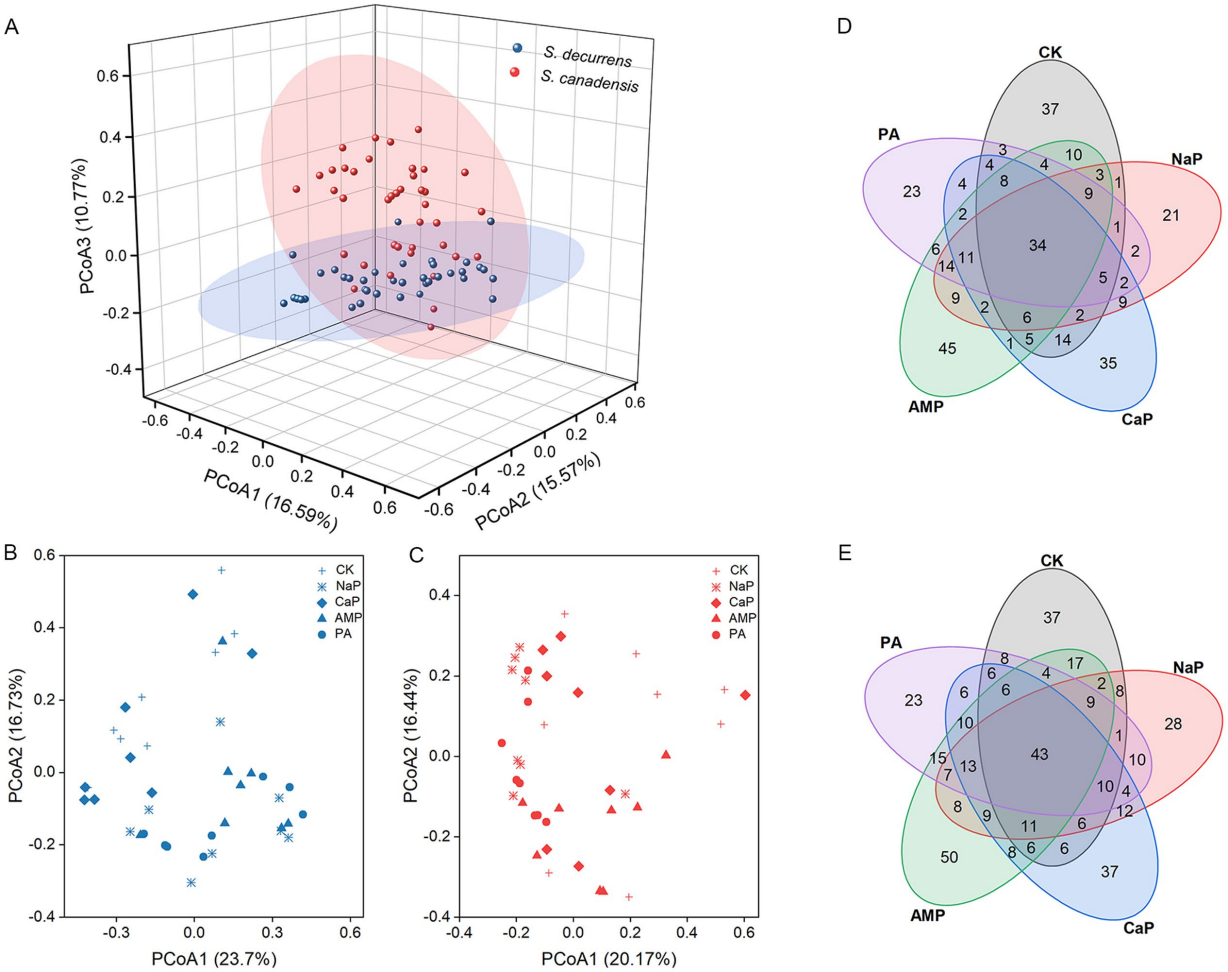
**(A)** Principal coordinates analysis (PCoA) based on the Bray–Curtis distance showing the differences in AMF community structure of *S. decurrens* and *S. canadensis*. Principal coordinates analysis (PCoA) based on the Bray–Curtis distance showing the difference in AMF community structure among five P treatments in **(B)** *S. decurrens* and **(C)** *S. canadensis*. CK: no P addition; NaP: sodium dihydrogen phosphate; CaP: hydroxyapatite; AMP: adenosine monophosphate; PA: myo-inositol hexakisphosphate. Venn diagrams showing the number of shared and unique ASVs among five P treatments in **(D)** *S. decurrens* and **(E)** *S. canadensis*. CK: no P addition; NaP: sodium dihydrogen phosphate; CaP: hydroxyapatite; AMP: adenosine monophosphate; PA: myo-inositol hexakisphosphate.

PCoA of Bray–Curtis distances revealed that AMF communities were delineated by plant species and forms of P (Figures 3A–C). In general, *S. decurrens* and *S. canadensis* harbored distinct AMF communities (Figure 3A; Supplementary Table S2). Adding P, except CaP, altered the AMF community structure of both *S. decurrens* and *S. canadensis* compared with the control treatment (Figures 3B,C; Supplementary Table S3). Moreover, the difference in AMF community composition between *S. decurrens* and *S. canadensis* intensified when the dominant P form in the soil changed (Supplementary Table S4). In addition, Venn plots showed that AMF communities in the two *Solidago* species had a greater proportion of common ASVs among different P forms (Figures 3D,E).

**Figure 4:**
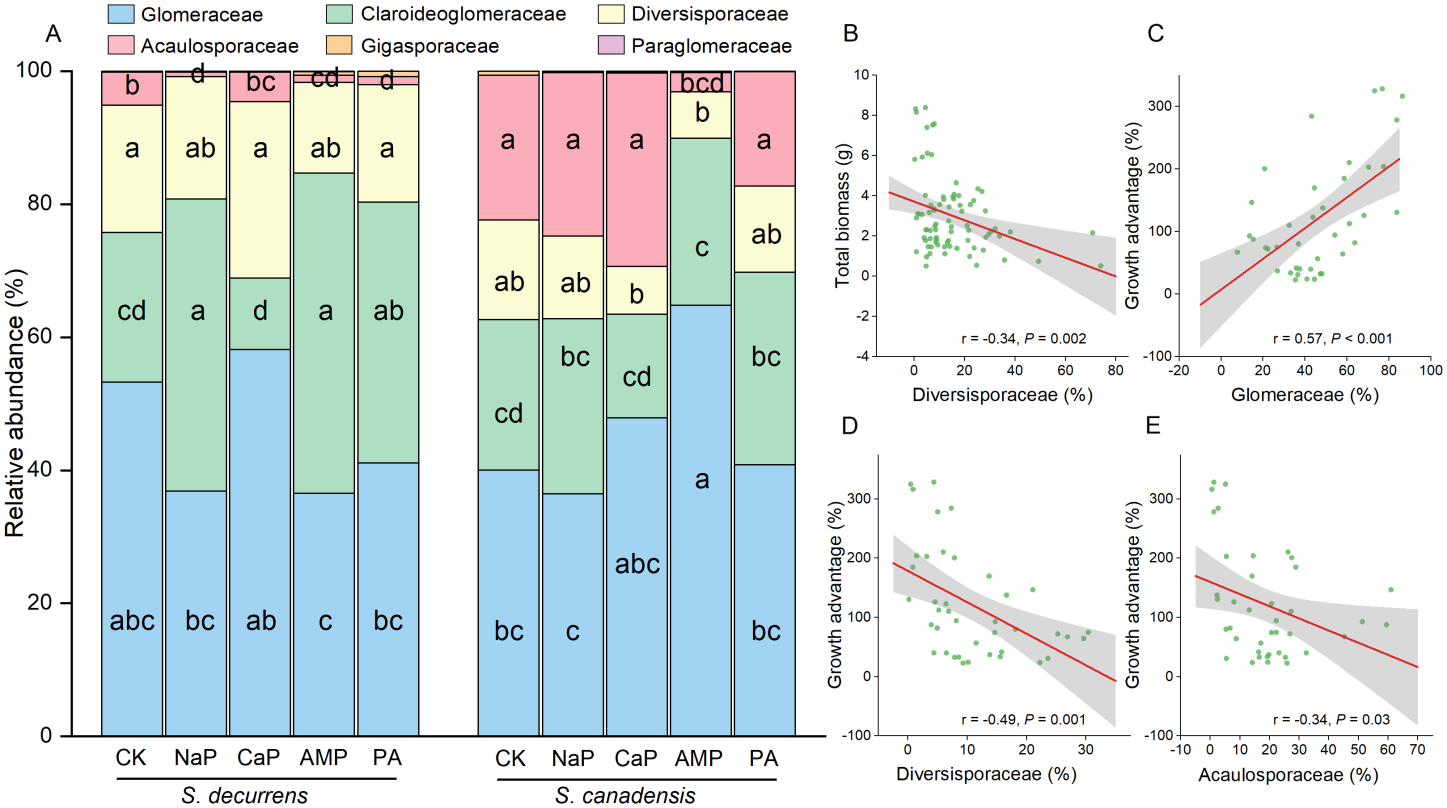
**(A)** Relative abundance of AMF at the family level of *S. decurrens* and *S. canadensis* under five P treatments. CK: no P addition; NaP: sodium dihydrogen phosphate; CaP: hydroxyapatite; AMP: adenosine monophosphate; PA: myo-inositol hexakisphosphate. The different lower-case letters among *S. decurrens* and *S. canadensis* denote significant differences at *p* < 0.05. **(B)** Pearson correlations between the relative abundance of Diversisporaceae and plant growth. Pearson correlations between the relative abundance of **(C)** Glomeraceae, **(D)** Diversisporaceae, and **(E)** Acaulosporaceae and the growth advantage of *S. canadensis* over *S. decurrens*. The gray ribbons represent the 95% confidence intervals.

ASVs detected in the rhizosphere soil were classified into six genera within six families. At the family level, the AMF communities were dominated by Glomeraceae (45.6%), followed by Claroideoglomeraceae (28.3%), Diversisporaceae (15.0%), Acaulosporaceae (10.8%), Gigasporaceae (0.27%) and Paraglomeraceae (0.05%) (Figure 4A). Overall, *S. decurrens* had higher relative abundances of Claroideoglomeraceae and Diversisporaceae and a lower relative abundance of Acaulosporaceae than *S. canadensis* (Figure 4A; Supplementary Table S5). P treatments resulted in distinct variations in AMF community structure between *S. decurrens* and *S. canadensis* (Table 1). For example, the applications of NaP and AMP increased Claroideoglomeraceae abundance but reduced Acaulosporaceae abundance relative to the control in *S. decurrens* (Figure 4A; Supplementary Table S5). AMP addition increased the relative abundance of Glomeraceae while decreasing the abundances of Diversisporaceae and Acaulosporaceae in *S. canadensis* (Figure 4A; Supplementary Table S5). Variations in AMF abundance were strongly correlated with plant performance. The relative abundance of Diversisporaceae was negatively correlated with whole-plant biomass (Figure 4B; Supplementary Table S6). The relative abundances of Glomeraceae were positively correlated with the growth advantage of *S.canadensis* over *S. decurrens* (Figure 4C; Supplementary Table S6), while those of Diversisporaceae and Acaulosporaceae were negatively correlated with the growth advantage of *S.canadensis* over *S. decurrens* (Figures 4D,E; Supplementary Table S6).

**Table 1:** Two-way analysis of variance (ANOVA) on the effects of plant species and P treatments on relative abundance of AMF at the family level.

Relative abundance (%)	Species (S)		Phosphorous (P)		S × P	
	*F*	*p*	*F*	*p*	*F*	*p*
Glomeraceae	0.03	0.87	1.59	0.19	2.34	0.06
Claroideoglomeraceae	**8.67**	**0.00**	**8.46**	**<0.001**	**2.82**	**0.03**
Diversisporaceae	**7.79**	**0.01**	1.02	0.41	1.05	0.39
Acaulosporaceae	**129.63**	**<0.001**	**10.80**	**<0.001**	**6.21**	**<0.001**
Gigasporaceae	0.00	0.99	0.38	0.82	1.70	0.16
Paraglomeraceae	0.08	0.78	0.89	0.48	0.86	0.49

Significant effects are shown in bold (*p* < 0.05).

### Arbuscular mycorrhizal fungi co-occurrence network and its relationship with plant performance

**Figure 5:**
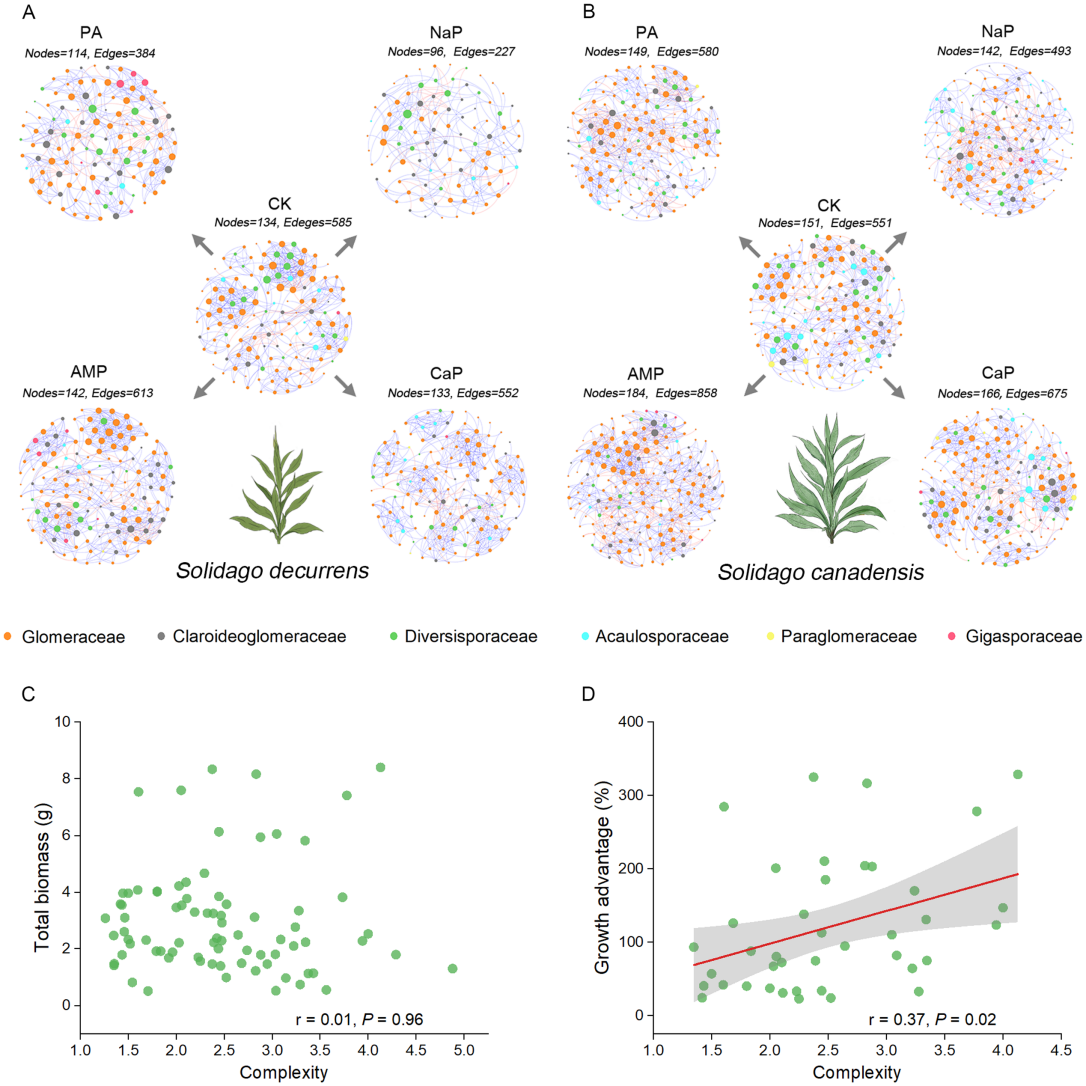
The AMF co-occurrence networks of **(A)** *S. decurrens* and **(B)** *S. canadensis* under five P treatments were visualized to show the significant associations (*r* > 0.50, *p* < 0.05) between AMF ASVs. CK: no P addition; NaP: sodium dihydrogen phosphate; CaP: hydroxyapatite; AMP: adenosine monophosphate; PA: myo-inositol hexakisphosphate. Each node represents an ASV, different colors represent different families, the node size represents the degree of the node, and edges denote significant correlations between ASVs (blue: positive correlation; red: negative correlation). Pearson correlations between the complexity of AMF co-occurrence networks and **(C)** plant growth, **(D)** growth advantage of *S. canadensis* over *S. decurrens*. The gray ribbons represent the 95% confidence intervals.

The AMF co-occurrence networks in *S. decurrens* and *S. canadensis* had distinct patterns with different P treatments (Figure 5; Table 2). In the control treatment, the average degree, average clustering coefficient, and complexity in the co-occurrence network of *S. decurrens* were greater than those of *S. canadensis* (Table 2). Adding P decreased average degree and complexity in the network of *S. decurrens*, while the opposite pattern was observed for *S. canadensis*, apart from with NaP (Table 2). Variations in complexity were positively correlated with the growth advantage of *S. canadensis* (Figure 5D).

**Table 2:** Topological features of the AMF co-occurrence network of two *Solidago* species under five P treatments.

Topological features	CK	NaP	CaP	AMP	PA
*S. decurrens*					
Nodes	134	96	133	142	114
Edge	585	227	552	613	384
Average degree	8.73	4.73	8.3	8.63	6.74
Average clustering coefficient	0.65	0.42	0.65	0.54	0.52
Positive correlations (%)	91.62	83.26	93.48	89.07	84.11
Negative correlations (%)	8.38	16.74	6.52	10.93	15.89
Density	0.07	0.05	0.06	0.06	0.06
Modularity	0.75	0.65	0.77	0.73	0.69
Complexity	4.37	2.36	4.15	4.32	3.37
Number of keystone species	4	3	2	5	9
*S. canadensis*					
Nodes	151	142	166	184	149
Edge	551	493	675	858	580
Average degree	7.30	6.94	8.13	9.33	7.79
Average clustering coefficient	0.53	0.48	0.54	0.52	0.55
Positive correlations (%)	91.10	77.28	87.41	78.09	73.97
Negative correlations (%)	8.89	22.72	12.59	21.91	26.03
Density	0.05	0.05	0.05	0.05	0.05
Modularity	0.74	0.64	0.73	0.66	0.70
Complexity	3.65	3.47	4.07	4.66	3.89
Number of keystone species	2	7	7	9	8

CK, no P addition; NaP, sodium dihydrogen phosphate; CaP, hydroxyapatite; AMP, adenosine monophosphate; PA, myo-inositol hexakisphosphate.

P treatment also had a significant impact on keystone species of the AMF co-occurrence networks (Supplementary Figure S1). The keystone species mainly belonged to the Glomeraceae, with a higher proportion in *S. canadensis* networks (76%) than those of *S. decurrens* (56%) (Supplementary Table S7). Relative to control, the number of keystone species decreased with inorganic P (i.e., NaP and CaP) but increased with organic P (i.e., AMP and PA) in the networks of *S. decurrens*. In contrast, the number of keystone species in *S. canadensis* networks was higher with all P treatments compared to the control, with the highest number of keystone species in the AMP treatment (Table 2). There was a strong correlation between the abundance of most keystone species and plant biomass and growth advantage of *S. canadensis* (Table 3). Interestingly, the ASVs that were positively correlated with plant biomass and growth advantage of *S. canadensis* all belonged to Glomeraceae, and the ASV that was negatively correlated with growth advantage of *S. canadensis* belonged to Claroideoglomeraceae (Table 3).

**Table 3:** Pearson correlations between keystone species abundance and plant growth, growth advantage of *S. canadensis*.

ASVID	Family	Total biomass		Growth advantage	
		*r*	*p*	*r*	*p*
ASV307	Glomeraceae	**0.30**	**0.01**	-	-
ASV721	Glomeraceae	**0.25**	**0.03**	-	-
ASV28	Glomeraceae	**0.44**	**<0.001**	**0.50**	**<0.001**
ASV95	Glomeraceae	**0.26**	**0.02**	0.19	0.25
ASV160	Glomeraceae	**0.26**	**0.02**	0.24	0.14
ASV240	Glomeraceae	**0.49**	**<0.001**	**0.47**	**0.002**
ASV354	Glomeraceae	**0.30**	**0.01**	**0.50**	**<0.001**
ASV609	Glomeraceae	**0.42**	**<0.001**	**0.32**	**0.045**
ASV830	Glomeraceae	**0.40**	**<0.001**	**0.42**	**0.008**
ASV1668	Glomeraceae	**0.28**	**0.01**	0.13	0.41
ASV63	Claroideoglomeraceae	−0.05	0.66	**−0.45**	**0.004**

Significant correlations are shown in bold (*p* < 0.05).

### Pathways linking P treatment and plant performance

**Figure 6:**
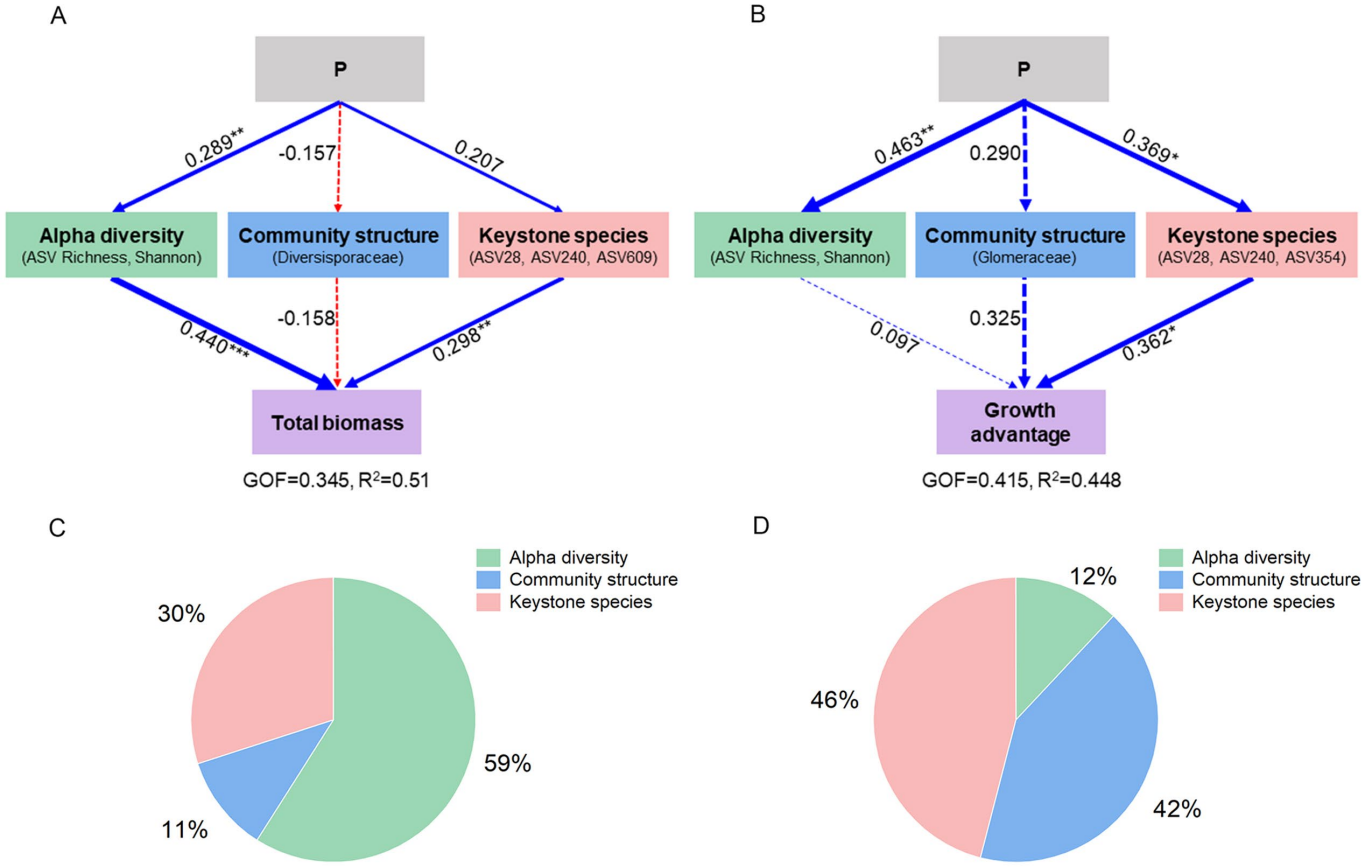
Path models showing the effects of P treatments on **(A)** plant growth and **(B)** growth advantage of *S. canadensis* over *S. decurrens* through alpha diversity, community structure and keystone species of the AMF. The line size represents the value of the path coefficient. The solid and dash lines represent significant and non-significant relationship. Blue lines represent positive effects, red lines represent negative effects. The asterisks denote the significance of path coefficient. *p < 0.05, **p < 0.01, ***p < 0.001. The final models fit the data well, as assessed using GOF and R^2^. The relative contribution of AMF latent variables to global explained observed variability (*R*^2^) of the **(C)** total biomass of two *Solidago* species and **(D)** growth advantage of *S. canadensis* over *S. decurrens.*

Overall, P treatments had a significant effect on plant performance *via* the modification of multiple AMF traits. P treatments favored plant species growth primarily through increasing alpha diversity of AMF (Figure 6A), which accounted for 59% of the total variance in plant biomass (Figure 6C). P treatments also enhanced the growth advantage of *S. canadensis* over *S. decurrens* through alterations in keystone species (Figure 6B); this factor had a greater impact on growth advantage than alpha diversity and community structure (Figure 6D).

## Discussion

Associations with AMF can influence host plants nutrient absorption and resistance to abiotic stress, further determining the invasion success of exotic plants (Zhang et al., 2017; Awaydul et al., 2019; Yu and He, 2022). In this study, by manipulating P in soils, we found that AMF diversity, community structure, and co-occurrence networks associated with native and invasive *Solidago* species responded distinctly to various P forms, indicating that soil nutrients are important drivers of symbiotic plant-fungal interactions in invasion ecology. P treatments may also affect the growth and invasiveness of invasive *Solidago*, as indicated by its growth advantage, through influencing alpha diversity and keystone taxa in the co-occurrence network of AMF. Together, our results suggest that differences in soil P may result in different interactions between AMF and native and invasive plants, and that multiple AMF traits play a vital role in enhancing *S. canadensis* invasion.

### Variations in plant–AMF interactions induced by soil P forms

Soil nutrients mediate interactions between symbiotic fungi and host plants (Qi et al., 2022; Wang et al., 2022), thus variations in the quality and quantity of soil nutrients may modify multiple AMF traits, consequently influencing plant performance. As expected, in this study, increased soil P availability affected the AMF community and promoted plant biomass, and this effect was strongly sensitive to P forms. Our results suggest that organic P facilitated greater AMF diversity and plant biomass than inorganic P for both native and invasive *Solidago* species. Moreover, even at the same level of P for a given species, multiple AMF characteristics, including diversity, certain AMF taxa, and topological features of the network varied distinctly to different organic and inorganic P forms. These results support our first hypothesis and the prevailing opinion that plant-fungal interactions are highly context-dependent.

Previous studies showed that plant-AMF interactions were either independent of invasive status (Bunn et al., 2015) or were greater (Sheng et al., 2022; Yu et al., 2022) or weaker (Vogelsang and Bever, 2009) in invaders than natives. In this study, we found that differences in plant-AMF interactions between *S. decurrens* and *S. canadensis* were amplified with greater P availability. Compared with *S. decurrens*, the AMF associated with *S. canadensis* responded to various P sources with greater plasticity in terms of diversity and community composition, potentially allowing *S. canadensis* to maintain a constant growth advantage across a variety of environments. We proposed a couple of scenarios to account for the divergence. First, native and invasive species exhibit a preference for different AMF taxa due to evolutionary and host filtering (Lekberg et al., 2013; Zhang et al., 2017), and may preferentially reward (e.g., provide carbohydrate or fatty acids) more beneficial microbial partners when faced with fluctuating soil nutrients, resulting in shifts in AMF communities (Johnson, 2010). Second, native and invasive plants grown in soil containing heterogeneous forms of P may differ in their plasticity to adjust root architecture and allocation (Yang et al., 2020; Zhang et al., 2022), which both govern interactions with AMF (Bergmann et al., 2020). Third, invasive species may also release greater concentrations of root chemical signaling compounds, such as flavonoids and strigolactones than native species, enhancing communications with mycorrhizal fungi (Inderjit et al., 2021; Yu et al., 2022).

### Linkages between AMF traits and plant performance

Microbial alpha diversity, a proxy for functional diversity, is susceptible to soil nutrients (Lang et al., 2022; Wang et al., 2022). In this study, we found a clear increase in AMF richness for *S. canadensis* grown in soil with AMP; these plants also had the highest biomass and growth advantage across all treatments. A greater AMF diversity normally means a stronger functional complementarity, such as occupying a broader resource niche (Loreau and Hector, 2001), and higher community-level soil acid phosphatase and alkaline phosphatase activity (Wang et al., 2014), allowing host plants to use resources more efficiently. In addition, diverse AMF can also enhance communication and cooperation with other microbes, such as phosphate-solubilizing bacteria and diazotrophs (Zhang et al., 2016; Zhu et al., 2018), which may increase P mineralization and nitrogen fixation. These benefits to host plants may determine the positive relationship between AMF diversity and plant growth. Moreover, our path analysis showed that AMF alpha diversity had a greater impact on plant performance than community structure or keystone species in the networks. Together, these results suggest that AMF alpha diversity could be a good predictor of plant growth and invasiveness for *S. canadensis* in a range of soil P environments.

Although there was an association between AMF diversity and plant performance, it should be noted that the non-negligible roles of certain AMF taxa on invasive plant-AMF interactions. For example, Sheng et al. (2022) found that a higher abundance of Glomeraceae in non-native *Conyza canadensis* enhanced their growth in invasive populations to a greater extent than native populations, suggesting specific AMF taxa play important roles in influencing the invasiveness of exotic species. Our results revealed that variations in abundance of Glomeraceae, the predominant taxon in the AMF families, exhibited positive relationship with the growth advantage of *S. canadensis* compared with Diversisporaceae and Acaulosporaceae. Normally, different AMF families have distinct functional traits, which can affect the amount and quality of benefits to their host plants. Previous studies found that Glomeraceae can colonize roots more rapidly, acquire and transport P to host plants more efficiently (de la Providencia et al., 2005; Voets et al., 2006), and grant better protection from pathogens (Powell et al., 2009) than other AMF families. This may partially explain why Glomeraceae appear to contribute substantially to the invasiveness of *S. canadensis*. Future works involving multiple pairs of invasive and native congeners are needed to better understand how Glomeraceae interact with invasive plants.

Soil microbes rarely exist independently; instead, they are connected to form complex ecological networks that may have a greater influence on plant performance and ecosystem functions than univariate diversity or composition metrics (Chen et al., 2022; Tian et al., 2022). This study demonstrated a positive correlation between AMF network complexity and growth advantage of *S. canadensis*; this is in accordance with a previous study reporting that mycorrhizal networks increased growth and nutrient acquisition of *S. canadensis* over that of native (Awaydul et al., 2019). This indicates that highly connected AMF co-occurrence networks favor invasive species over native species. In addition to network complexity, keystone species in the networks, identified using network topological features, also play an important role in maintaining greater AMF mutualisms on *S. canadensis* than *S. decurrens*. We found that keystone species positively linked to plant biomass occurred more frequently in the AMF network of *S. canadensis* compared to native species. Moreover, the significant relationship between keystone species and growth advantage of *S. canadensis* remained robust when the confounding factors (i.e., alpha diversity and community structure) were controlled for in the path analysis. In addition, all the keystone species that were positively correlated with plant biomass and growth advantage in *S. canadensis* belonged to Glomeraceae, an AMF family that has been reported to enhance invasive plant growth previously (Sheng et al., 2022). Thus, the more substantial effects of keystone species on the growth advantage in *S. canadensis*, relative to other AMF traits, implies that the symbiotic fungus-mediated invasiveness of exotic species is likely enhanced by key individual taxa rather than the whole community.

Together, these findings supported our second hypothesis that a variety of AMF traits induced by different soil P sources contribute differently to plant growth and growth advantage in *S. canadensis*. However, it should be noted that other mycorrhizal traits, such as mycorrhizal colonization and hyphal length density, may also contribute to the superior performance of invasive species. Thus, our work highlights the necessity to disentangle the effects of multiple mycorrhizal traits on plant invasion in further studies. Furthermore, although this study focused primarily on the effect of different forms of P on invasive plant-AMF interactions, the soil nutrient availability may also have a role (Chen et al., 2020). Notwithstanding these points, our findings may have some implications for exotic species management strategies, and could improve understanding of the microbiome-related mechanisms underpinning the invasion success of exotic plants for a number of reasons. First, P fractions vary by ecosystem (Song et al., 2007; Azene et al., 2022). We found that organic P facilitated growth of the invasive *Solidago* to a greater extent than inorganic P, which is in accordance with previous studies (Yang et al., 2020; Zhang et al., 2022). Accordingly, ecosystems with higher organic P might carry a higher risk of plant invasion. Second, microbial-based invasive plant management has been suggested as a novel tool for controlling invasive plants (Shahrtash and Brown, 2021). In this study, keystone species had the strongest effect on the growth advantage of *S. canadenis*, which means keystone species play a potential functional role in plant invasion. This is consistent with a recent study that reported keystone species contributed to invader tolerance to biotic stressors (Xu et al., 2022). Therefore, identifying keystone species that disrupt nutrient absorption and facilitate disease development in invasive plants could enable a microbial invasive plant management strategy. Finally, AMF enable a better performance of invader over congeneric native species through multiple dimension traits, which previous studies have not investigated. Future studies should address the correlations between different AMF traits and plant invasions.

In summary, our results show that plant growth, growth advantage of invasive species, and AMF traits vary with the form of soil P. Furthermore, differences in AMF traits with different sources of P have different effects on plant growth and growth advantage of *S. canadensis.* These results indicate that the AMF community mediates plant growth and growth advantage under different nutrient conditions, highlighting the importance of environmental context on plant invasions. They also emphasize the relative contribution of microbial community composition and their interconnections in enhancing the environmental adaptability of invasive plants. A more detailed understanding of the mechanism by which AMF communities are altered depending on the P forms in the soil will help guide invasive plant management. Future experiments that simultaneously consider AMF community, mycorrhizal association and hyphal density will contribute to a better understanding of the role of soil microbes and nutrients in invasion success of exotic species.

## Data availability statement

The datasets presented in this study can be found in online repositories. The names of the repository/repositories and accession number(s) can be found at: https://www.ncbi.nlm.nih.gov/, PRJNA922727.

## Author contributions

HY designed the study. LC, MW, PM, and YX conducted the experiments. LC and HY analyzed the data and wrote the manuscript. JD and YS revised the manuscript. All authors discussed the results and approved the final manuscript.

## Funding

This work was supported by the National Natural Science Foundation of China (32001233 and U21A20190), and the China Postdoctoral Science Foundation (2019 M662526).

## Conflict of interest

The authors declare that the research was conducted in the absence of any commercial or financial relationships that could be construed as a potential conflict of interest.

## Publisher’s note

All claims expressed in this article are solely those of the authors and do not necessarily represent those of their affiliated organizations, or those of the publisher, the editors and the reviewers. Any product that may be evaluated in this article, or claim that may be made by its manufacturer, is not guaranteed or endorsed by the publisher.

## Footnotes

^1^http://mem.rcees.ac.cn:8081
