# Supplementary material for: Soil phosphorus form affects the advantages that arbuscular mycorrhizal fungi confer on the invasive plant species, Solidago canadensis, over its congener
Source: Front Microbiol. 2023 Apr 14;14:1160631. doi: 10.3389/fmicb.2023.1160631 (PMC10140316; doi:10.3389/fmicb.2023.1160631)

**Supplementary Material**

**Table S1** Dillon–Goldstein’s rho (DG.rho) for a latent variable and loading of each manifest variable for its latent variable in the partial least squares path modeling for plant growth and the growth advantage of *S. canadensis*.

| Path models | Latent variables | Manifest variables | DG.rho | Loading |
| --- | --- | --- | --- | --- |
| Total biomass  (GoF:0.345  R^2^:0.510) | P | Ranking of P use efficacy | 1 | 1 |
|  | Alpha diversity | Richness | 0.937 | 0.967 |
|  |  | Shannon |  | 0.903 |
|  | Community structure | Diversisporaceae | 1 | 1 |
|  | Keystone species | ASV609 | 0.832 | 0.763 |
|  |  | ASV240 |  | 0.839 |
|  |  | ASV28 |  | 0.760 |
|  | Total biomass | Biomass | 1 | 1 |
| Growth advantage (GoF:0.415  R^2^:0.448) | P | Ranking of P use efficacy | 1 | 1 |
|  | Alpha diversity | Richness | 0.940 | 0.952 |
|  |  | Shannon |  | 0.931 |
|  | Community structure | Glomeraceae | 1 | 1 |
|  | Keystone species | ASV354 | 0.879 | 0.923 |
|  |  | ASV240 |  | 0.844 |
|  |  | ASV28 |  | 0.750 |
|  | Growth advantage | Growth advantage | 1 | 1 |

**Table S2** Dissimilarity analysis (PERMANOVA) of AMF community under five P treatments across two *Solidago* species*.* Significant effects are shown in bold (*P* < 0.05).

| Dissimilarity based on Bray-Curtis | PERMANOVA | |
| --- | --- | --- |
|  | *F* | *P* |
| Species (S) | **2.59** | **<0.001** |
| Phosphorus (P) | **1.60** | **<0.001** |
| S × P | 1.01 | 0.460 |

**Table S3** Dissimilarity analysis (PERMANOVA) of AMF community under five P treatments across *S. decurrens and S. canadensis*. CK: no P addition; NaP: sodium dihydrogen phosphate; CaP: hydroxyapatite; AMP: adenosine monophosphate; PA: myo-inositol hexakisphosphate. Significant effects are shown in bold (*P* < 0.05).

| Species | Dissimilarity based on Bray-Curtis |  | PERMANOVA | |
| --- | --- | --- | --- | --- |
|  |  |  | *F* | *P* |
| *S. decurrens* | CK vs NaP |  | **3.10** | **0.001** |
|  | CK vs CaP |  | 0.94 | 0.48 |
|  | CK vs AMP |  | **2.68** | **0.003** |
|  | CK vs PA |  | **3.51** | **0.002** |
| *S. canadensis* | CK vs NaP |  | **2.65** | **0.005** |
|  | CK vs CaP |  | 0.85 | 0.61 |
|  | CK vs AMP |  | **2.27** | **0.01** |
|  | CK vs PA |  | **3.11** | **0.006** |

**Table S4** Dissimilarity analysis (PERMANOVA) of AMF communities of *S. decurrens and S. canadensis* under five P treatments. CK: no P addition; NaP: sodium dihydrogen phosphate; CaP: hydroxyapatite; AMP: adenosine monophosphate; PA: myo-inositol hexakisphosphate. Significant effects are shown in bold (*P* < 0.05).

| Phosphorous (P) | Dissimilarity based on Bray-Curtis |  | PERMANOVA | |
| --- | --- | --- | --- | --- |
|  |  |  | *F* | *P* |
| CK | *S. decurrens* vs *S. canadensis* |  | 0.98 | 0.44 |
| NaP | *S. decurrens* vs *S. canadensis* |  | **3.70** | **0.00** |
| CaP | *S. decurrens* vs *S. canadensis* |  | **2.36** | **0.01** |
| AMP | *S. decurrens* vs *S. canadensis* |  | **2.40** | **0.01** |
| PA | *S. decurrens* vs *S. canadensis* |  | **3.91** | **0.00** |

**Table S5** One-way analysis of variance (ANOVA) for the relative abundance of AMF at the family level. CK: no P addition; NaP: sodium dihydrogen phosphate; CaP: hydroxyapatite; AMP: adenosine monophosphate; PA: myo-inositol hexakisphosphate.

| AMF family | *S. decurrens* | | | | |  | *S. canadensis* | | | | |
| --- | --- | --- | --- | --- | --- | --- | --- | --- | --- | --- | --- |
|  | CK | NaP | CaP | AMP | PA |  | CK | NaP | CaP | AMP | PA |
| Glomeraceae | 53.3± 6.5abc | 36.8 ± 10.0bc | 58.2 ± 8.9ab | 36.5± 7.8c | 41.2 ± 7.6bc |  | 40.0 ± 6.5bc | 36.5 ± 8.3c | 47.9 ± 8.2abc | 64.8 ± 6.8a | 40.9 ± 2.5bc |
| Claroideoglomeraceae | 22.5 ± 4.3cd | 44.0 ± 9.0a | 10.7 ± 2.7d | 48.2 ± 6.8a | 39.2 ± 4.6ab |  | 22.6 ± 4.1cd | 26.3 ± 3.6bc | 15.6 ± 3.6cd | 25.1 ± 4.7c | 28.9 ± 2.1bc |
| Diversisporaceae | 19.1 ± 4.3a | 18.4 ± 8.6ab | 26.5 ± 8.4a | 13.6 ± 3.1ab | 17.6 ± 3.4a |  | 15.0 ± 3.5ab | 12.4 ± 3.3ab | 7.2 ± 2.4b | 7.0 ± 2.4b | 12.9 ± 2.7ab |
| Acaulosporaceae | 5.0 ± 1.1b | 0.7 ± 0.3d | 4.4 ± 1.5bc | 1.1 ± 0.3cd | 1.2 ± 0.9d |  | 21.8 ± 4.8a | 24.5 ± 4.4a | 29.1 ± 7.2a | 2.9 ± 0.7bcd | 17.2 ± 2.4a |
| Gigasporaceae | 0.06 ± 0.05 | 0.1 ± 0.1 | 0.06 ± 0.05 | 0.5 ± 0.4 | 0.8 ± 0.6 |  | 0.6 ± 0.3 | 0.2 ± 0.1 | 0.1 ± 0.08 | 0.2 ± 0.1 | 0.02 ± 0.02 |
| Paraglomeraceae | 0.07 ± 0.07 | 0.06 ± 0.03 | 0.06 ± 0.05 | 0.06 ± 0.03 | 0 |  | 0.01 ± 0.01 | 0.06 ± 0.03 | 0.2 ± 0.1 | 0.01±0.01 | 0.03 ± 0.03 |

Values are means ± SE (n = 8). Different letters in the same row indicate a significant difference at *P* < 0.05.

**Table S6** Pearson correlations between the relative abundance of AMF at the family level and plant growth, growth advantage of *S. canadensis* over *S. decurrens.* Significant correlations are shown in bold (*P* < 0.05).

| Relative abundance (%) | Total biomass | |  | Growth advantage | |
| --- | --- | --- | --- | --- | --- |
|  | *r* | *P* |  | *r* | *P* |
| Glomeraceae | 0.17 | 0.12 |  | **0.57** | **<0.001** |
| Claroideoglomeraceae | -0.008 | 0.94 |  | -0.25 | 0.11 |
| Diversisporaceae | **-0.34** | **0.002** |  | **-0.49** | **0.001** |
| Acaulosporaceae | 0.07 | 0.54 |  | **-0.34** | **0.03** |
| Gigasporaceae | -0.04 | 0.74 |  | 0.06 | 0.73 |
| Paraglomeraceae | -0.07 | 0.56 |  | 0.10 | 0.54 |

**Table S7** AMF community composition of the keystone species of two *Solidago* species under five P treatments. CK: no P addition; NaP: sodium dihydrogen phosphate; CaP: hydroxyapatite; AMP: adenosine monophosphate; PA: myo-inositol hexakisphosphate.

| Treatment | ASVID | Category | Family | Genus |
| --- | --- | --- | --- | --- |
| *S. decurrens* |  |  |  |  |
| CK | ASV31 | Connector hubs | Glomeraceae | *Glomus* |
|  | ASV405 | Connector hubs | Glomeraceae | *Glomus* |
|  | ASV616 | Connector hubs | Glomeraceae | *Glomus* |
|  | ASV90 | Connector hubs | Glomeraceae | *Glomus* |
| NaP | ASV122 | Connector hubs | Diversisporaceae | *Diversispora* |
|  | ASV151 | Connector hubs | Claroideoglomeraceae | *Claroideoglomus* |
|  | ASV721 | Connector hubs | Glomeraceae | *Glomus* |
| CaP | ASV202 | Connector hubs | Glomeraceae | *Glomus* |
|  | ASV6 | Connector hubs | Claroideoglomeraceae | *Claroideoglomus* |
| AMP | ASV126 | Connector hubs | Glomeraceae | *Glomus* |
|  | ASV181 | Connector hubs | Glomeraceae | *Glomus* |
|  | ASV390 | Connector hubs | Claroideoglomeraceae | *Claroideoglomus* |
|  | ASV392 | Connector hubs | Glomeraceae | *Glomus* |
|  | ASV368 | Module hubs | Acaulosporaceae | *Acaulospora* |
| PA | ASV109 | Connector hubs | Glomeraceae | *Glomus* |
|  | ASV122 | Connector hubs | Diversisporaceae | *Diversispora* |
|  | ASV126 | Connector hubs | Glomeraceae | *Glomus* |
|  | ASV141 | Connector hubs | Diversisporaceae | *Diversispora* |
|  | ASV201 | Connector hubs | Diversisporaceae | *Diversispora* |
|  | ASV238 | Connector hubs | Claroideoglomeraceae | *Claroideoglomus* |
|  | ASV3 | Connector hubs | Glomeraceae | *Glomus* |
|  | ASV307 | Connector hubs | Glomeraceae | *Glomus* |
|  | ASV276 | Module hubs | Claroideoglomeraceae | *Claroideoglomus* |
| *S. canadensis* |  |  |  |  |
| CK | ASV3 | Connector hubs | Glomeraceae | *Glomus* |
|  | ASV119 | Module hubs | Glomeraceae | *Glomus* |
| NaP | ASV126 | Connector hubs | Glomeraceae | *Glomus* |
|  | ASV1538 | Connector hubs | Glomeraceae | *Glomus* |
|  | ASV1668 | Connector hubs | Glomeraceae | *Glomus* |
|  | ASV473 | Connector hubs | Glomeraceae | *Glomus* |
|  | ASV63 | Connector hubs | Claroideoglomeraceae | *Claroideoglomus* |
|  | ASV830 | Connector hubs | Glomeraceae | *Glomus* |
|  | ASV95 | Connector hubs | Glomeraceae | *Glomus* |
| CaP | ASV109 | Connector hubs | Glomeraceae | *Glomus* |
|  | ASV163 | Connector hubs | Diversisporaceae | *Diversispora* |
|  | ASV570 | Connector hubs | Glomeraceae | *Glomus* |
|  | ASV608 | Connector hubs | Glomeraceae | *Glomus* |
|  | ASV63 | Connector hubs | Claroideoglomeraceae | *Claroideoglomus* |
|  | ASV66 | Connector hubs | Diversisporaceae | *Diversispora* |
|  | ASV72 | Connector hubs | Diversisporaceae | *Diversispora* |
| AMP | ASV181 | Connector hubs | Glomeraceae | *Glomus* |
|  | ASV240 | Connector hubs | Glomeraceae | *Glomus* |
|  | ASV354 | Connector hubs | Glomeraceae | *Glomus* |
|  | ASV560 | Connector hubs | Glomeraceae | *Glomus* |
|  | ASV574 | Connector hubs | Glomeraceae | *Glomus* |
|  | ASV63 | Connector hubs | Claroideoglomeraceae | *Claroideoglomus* |
|  | ASV773 | Connector hubs | Glomeraceae | *Glomus* |
|  | ASV30 | Module hubs | Claroideoglomeraceae | *Claroideoglomus* |
|  | ASV597 | Module hubs | Glomeraceae | *Glomus* |
| PA | ASV160 | Connector hubs | Glomeraceae | *Glomus* |
|  | ASV28 | Connector hubs | Glomeraceae | *Glomus* |
|  | ASV31 | Connector hubs | Glomeraceae | *Glomus* |
|  | ASV354 | Connector hubs | Glomeraceae | *Glomus* |
|  | ASV560 | Connector hubs | Glomeraceae | *Glomus* |
|  | ASV609 | Connector hubs | Glomeraceae | *Glomus* |
|  | ASV704 | Connector hubs | Glomeraceae | *Glomus* |
|  | ASV872 | Module hubs | Diversisporaceae | *Diversispora* |

**Figure S1** Within-module (*Zi*) and among-module (*Pi*) connectivity plots showing the distribution of AMF based on their topological roles of *S. decurrens* under (A) CK, (B) NaP, (C) CaP, (D) AMP, and (E) PA, and of *S. canadensis* under (F) CK, (G) NaP, (H) CaP, (I) AMP, and (J) PA. CK: no P addition; NaP: sodium dihydrogen phosphate; CaP: hydroxyapatite; AMP: adenosine monophosphate; PA: myo-inositol hexakisphosphate.
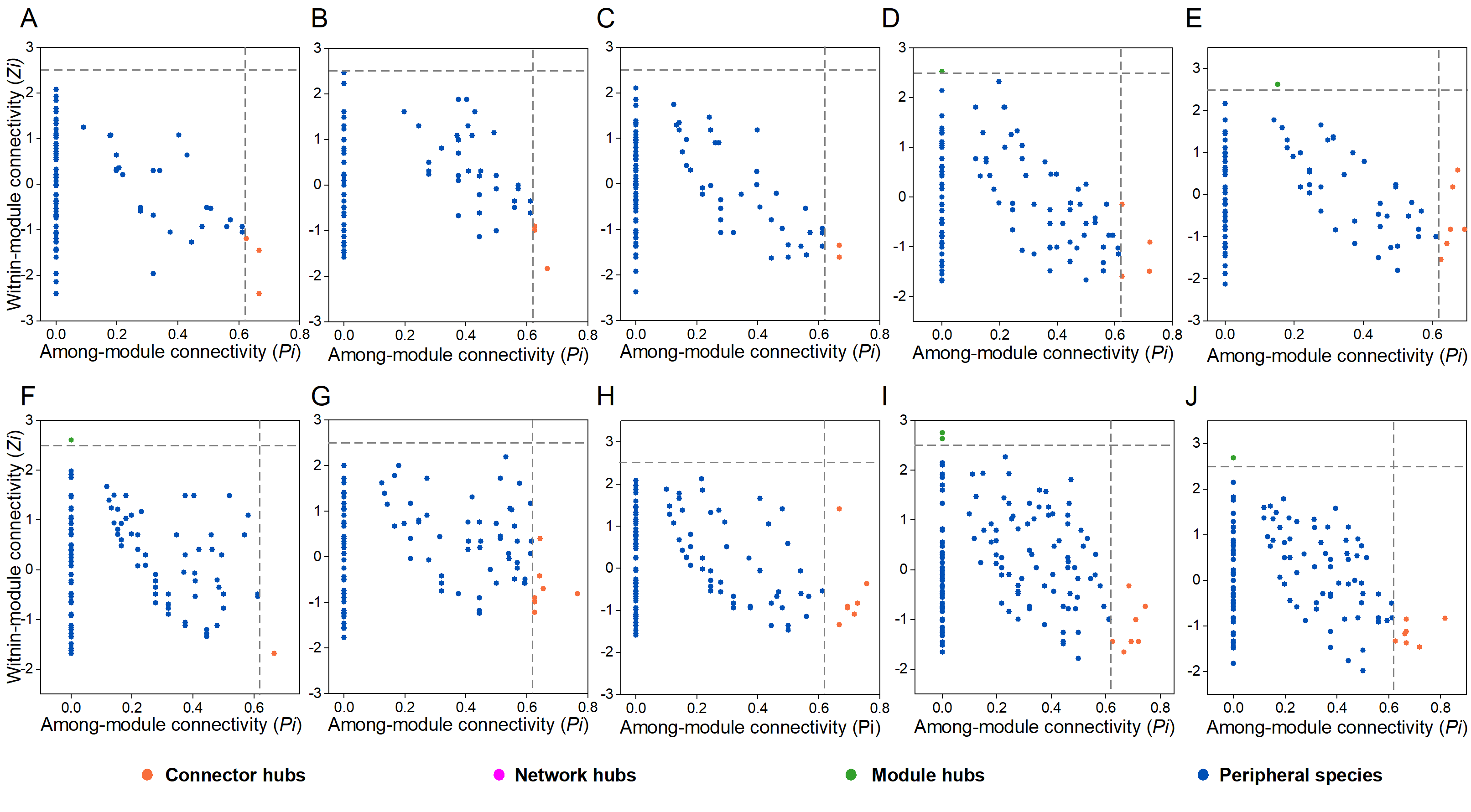

Supplement: Supplementary file 1 [file Data_Sheet_1.docx]
